# Professional Identity, Educator Competence Perception and Willingness to Engage in Geriatric Nursing Among Nursing Students: A Cross‐Sectional Study

**DOI:** 10.1002/nop2.70804

**Published:** 2026-09-28

**Authors:** Ting Liao, Jinmei Zou, Min Wang, Yuqing Song, Li Cai, Jin Yin, Yao Chen, Yating Wang

**Affiliations:** ^1^ School of Nursing Sichuan Vocational College of Health and Rehabilitation Zigong China; ^2^ Department of Nursing West China Second University Hospital of Sichuan University Chengdu China

**Keywords:** geriatric nursing, gerontological nurse educator, nursing students, professional identity, willingness

## Abstract

**Aim:**

To investigate the interrelationships of professional identity, educator competence perception and willingness to engage in geriatric nursing among Chinese nursing students.

**Design:**

A cross‐sectional study design.

**Methods:**

This study involved a cross‐sectional descriptive study. A total of 842 nursing students enrolled in online learning were included in this study. We used the Professional Identity Questionnaire for Nursing Students (PIQNS), the Gerontological Nurse Teacher Scale (GeNTS), and the Willingness Engaging in Aged Nursing Scale (WEANS) to measure levels of PI, educator competence perception and willingness to engage in geriatric nursing. The structural equation model and bootstrap method were used to identify the association and interaction among the three variables.

**Results:**

The scores of nursing students' PI, educator competence perception and willingness to engage in geriatric nursing were (59.77 ± 10.85), (111.70 ± 19.42), and (71.71 ± 11.17), respectively. After adjusting for age, gender, and academic year, PI was positively associated with educator competence perception (*β* = 0.70, *p* < 0.001), and educator competence perception was positively associated with willingness to engage in geriatric nursing (*β* = 0.33, *p* < 0.001). In a cross‐sectional mediational model, educator competence perception accounted for an indirect association between PI and willingness (standardised indirect effect = 0.230, 95% bootstrap CI [0.164, 0.296]); after model adjustment and controlling for age, gender, and grade, the final structural model yielded good fit (*χ* ^2^/df = 2.954, *p* < 0.001, GFI = 0.961, TLI = 0.977, CFI = 0.983, NFI = 0.975, RMSEA = 0.048).

**Conclusion:**

Professional identity of nursing students and perceived educator competence are positively associated with students' willingness to engage in geriatric nursing. Educational interventions may focus on these areas to address the growing need for geriatric care professionals.

**Patient or Public Contribution:**

No patient or public contribution.

**Abbreviations**
AVE: average variance extracted
CFA: confirmatory factor analysis
CFI: comparative fit index
CI: confidence interval
CR: composite reliability
GeNTS: Gerontological Nurse Teacher Scale
GFI: goodness‐of‐fit index
HTMT: heterotrait‐monotrait ratio of correlations
NFI: normed fit index
PI: professional identity
PIQNS: Professional Identity Questionnaire for Nursing Students
RMSEA: root mean square error of approximation
SD: standard deviation
SE: standard error
SEM: structural equation modelling
SRMR: standardised root mean square residual
TLI: Tucker‐Lewis index
WEANS: Willingness Engaging in Aged Nursing Scale
*α*
: Cronbach's alpha
*β*
: standardised path coefficient

## 1 Introduction

The global aging population increase the need for geriatric nurses (Cesari et al. 2022). Nevertheless, nursing students show limited interest in pursuing careers in geriatric nursing (Koskinen et al. 2022). In China, only 38.1% of nursing students express a willingness to work with older people after graduation (Guo et al. 2021; Yan et al. 2022). This shortage of qualified geriatric nurses has become a critical barrier to meeting elderly care needs (Qumer and Fatima 2023). Identifying modifiable factors that improve students' willingness to engage in geriatric nursing is urgently needed for nursing education and practice.

Professional identity (PI) is a core factor shaping nursing students' career willingness (Wei et al. 2021). Strong PI enhances career commitment and persistence, which are crucial in geriatric nursing due to its need for patience and specialised skills (Bahadir‐Yilmaz 2018). Educator competence is another key environmental factor that influences students' professional development (Garbrah, Kankkunen, and Välimäki 2020). Most existing studies focus on the link between PI and career willingness. However, the role of educator competence perception in PI and career willingness has not been well explored. In addition, studies among vocational college nursing students, who represent a primary source of frontline geriatric nurses, remain insufficiently explored.

This study aimed to explore the relationship between PI, perceived educator competence, and willingness to engage in geriatric nursing among vocational college nursing students.

## 2 Backgrounds

Nursing students' willingness to pursue a career in geriatric nursing is influenced by numerous factors (Okuyan et al. 2020). Existing studies (Cheng 2021; Henderson et al. 2012; Jiang and Jin 2022) revealed that nursing students' career willingness was influenced by various determinants, including positive attitudes and knowledge about aging, a supportive learning atmosphere, the quality of contact with older adults, personal experiences and values, job prospects, and societal attitudes toward aging and older adults, which also play a significant role in shaping students' career choices. Additionally, professional identity (Arreciado Marañón and Isla Pera 2015) and gerontological nurse educator's competence (Bahadir‐Yilmaz 2018) affect students' willingness to care for older adults.

Professional identity (PI) serves as a cornerstone for nursing students' engagement in geriatric care and is shaped throughout their educational and practical journey (Fitzgerald and Clukey 2021). It is a dynamic and continuous developmental process that forms during geriatric nursing education and evolves with clinical practice and professional socialisation. Developing a robust geriatric nursing professional identity is vital for nursing students, as it fosters their career commitment, learning motivation, and long‐term persistence in the geriatric care field (Zhao et al. 2022). Previous studies have highlighted that professional identity is closely linked to nursing students' career willingness (Rosario 2022), professional values, and positive attitudes toward older adults (Chai et al. 2019).

Educator competence perception refers to nursing students' subjective evaluation of the professional knowledge, teaching skills, practical guidance, and professional ethics demonstrated by geriatric nursing educators (Garbrah, Kankkunen, and Välimäki 2020; Liao, Wang, and Zou 2024). This perception is formed as students engage in geriatric nursing courses and clinical placements, and it is continually refined through teaching interactions, role modelling, and practical guidance from educators (Mattsson and Rosendahl 2022). Nursing students who hold positive perceptions of educator competence are more likely to form positive attitudes toward working with older adults (Tohmola et al. 2021). Importantly, positive educator competence perception strongly predicts students' learning motivation, professional confidence, and career commitment, and it serves as a key environmental factor that directly promotes nursing students' willingness to engage in geriatric nursing (Garbrah, Kankkunen, and Välimäki 2020).

Overall, the development of PI and educator competence perception is not only a core component of geriatric nursing education but also serves a pivotal function in shaping nursing students' career intentions. For example, cross‐sectional studies involving Chinese nursing students have verified that professional identity is significantly and positively associated with their willingness to provide care for older adults (Moll‐Jongerius et al. 2023). Other studies (Garbrah, Kankkunen, and Välimäki 2020; Chai et al. 2019) have also demonstrated that students' positive perception of geriatric nursing educators' competence is closely linked to stronger career intentions in geriatric care. Moreover, professional identity has been found to promote a more positive evaluation of educator competence among nursing students (Vabo et al. 2021).

Although the significance of professional identity in influencing nursing students' willingness to engage in geriatric nursing is well acknowledged, the underlying mechanisms remain insufficiently explored. The mediating role of educator competence perception in the relationship between PI and willingness to engage in geriatric nursing has not been well explored. Thus, this study aimed to explore the mediating role of educator competence perception in the relationship between PI and willingness to engage in geriatric nursing. It seeks to explore the pathway of each factor to nursing students' geriatric care career intentions. By offering valuable insights into the determinants of geriatric nursing willingness, the findings of this study are expected to inform the development of evidence‐based strategies that improve geriatric nursing education.

### 2.1 Theoretical Framework

The conceptual model for this study is derived from social cognitive theory (Lent et al. 1994), which posits that nursing students' PI directly influences their choice of geriatric nursing‐related careers. Additionally, the model suggests that social support (from school, family and peers) may further strengthen or weaken the influence of PI on career choice. In this study, students' perception of gerontological nursing faculty competence is considered a form of social support. It is hypothesised that this perception can strengthen the effect of nursing students' self‐efficacy on career choice outcomes.

### 2.2 Study Aim and Hypotheses

The aim of this study was to assess the current status of PI, educator competence perception and willingness to engage in elderly nursing among nursing students. We also tried to clarify the interaction among these three variables and the mediating effect of educator competence perception between PI and willingness to engage in elderly nursing.

This study hypothesised that (1) Students' PI influences their willingness to engage in elderly nursing. (2) PI influences their willingness to engage in elderly nursing through the mediating effect of educator competence perception.

## 3 Methods

### 3.1 Research Design

This study used a cross‐sectional study design to evaluate PI, educator competence perception and willingness to engage in geriatric nursing among nursing students, and to explore the pathway of PI and educator competence perception to students' willingness to engage in geriatric nursing based on social cognitive theory. This study was conducted following the Strengthening the Reporting of Observational Studies in Epidemiology (STROBE) statement (Elm et al. 2007).

### 3.2 Participants

We used convenience sampling to recruit participants from the nursing department of a vocational college in Sichuan, China. This sampling method was selected for its practicality and efficiency in accessing a suitable group of participants within a limited timeframe. It enabled timely data collection and reflected the real status of vocational nursing students (Ahmed 2024).

An a priori calculation of the sample size was conducted based on the study by Sim et al. (2021), which indicated that at least 620 participants were required to construct a structural equation model with the smallest medium indirect effect size (0.4), four indicators and 0.4 loadings, using the bootstrap method. To account for potential non‐responses, an 11.1% non‐response rate was added, following a previous cross‐sectional study involving 544 Chinese nursing students (Zeng et al. 2019). Therefore, a minimum of 690 participants was determined to be necessary.

The inclusion criteria were: (1) full‐time nursing students, (2) completing a geriatric nursing course, and (3) voluntary participation in this study. We excluded students who left due to illness or personal reasons.

### 3.3 Data Collection

An online survey was administered via the Wenjuanxing platform from April to September 2024. Class monitors distributed the survey link (https://www.wjx.cn/vm/YqVT2yk.aspx) to their respective WeChat groups to avoid any coercion from teachers. Participants were informed about the purpose and content of this study. Participation was voluntary, and the survey results did not impact academic performance.

### 3.4 Ethics Approval and Consent to Participate

The study was conducted in accordance with the Declaration of Helsinki and approved by the Zigong First People's Hospital Committee (No. Ethics (Research) No. 129 of 2023). Before accessing the formal questionnaire, all participants were required to read a digital informed consent form. Participants who clicked the “Agree and Continue” button could enter the formal survey, ensuring voluntary and informed participation.

### 3.5 Measures

*The Professional Identity Questionnaire for Nursing Students (PIQNS)* compiled by Hao et al. (2014) was used to assess the professional identity of the subjects. The PIQNS consists of 17 items, categorised into five dimensions: professional self‐image (6 items), benefits of retention and risk of turnover (4 items), social comparison and self‐reflection (3 items), independence of career choice (2 items), and social modelling (2 items). Respondents rate each item on a 5‐point scale, ranging from 1 (‘not true at all’) to 5 (‘true nearly all the time’). Higher scores indicate a higher degree of PI experienced by the participants. The PIQNS has shown good internal consistency among Chinese nursing college students, with a Cronbach's *α* value of 0.827 for the total scale (Hao et al. 2014). In our study, the Cronbach's *α* value for the PIQNS was 0.891.

*The Gerontological Nurse Teacher Scale (GeNTS)* was proposed in 2020 by Garbrah, Kankkunen, and Vlimki (2020) and later translated and revised into Chinese by our research team (Liao, Wang, and Zou 2024). The Chinese version consists of 30 items across five dimensions: theoretical knowledge (7 items), student care (5 items), professional enthusiasm (6 items), practical training (6 items), and social ethics (6 items). Items are assessed using a 5‐point scoring system ranging from 1 (completely disagree) to 5 (completely agree), with higher scores indicating a more positive evaluation of the gerontological nurse teacher's competencies (Liao, Wang, and Zou 2024). The Cronbach's alpha of GENTS was 0.977, indicating good reliability. In our study, the Cronbach's *α* value for the GeNTS was 0.960.

*The Willingness Engaging in Aged Nursing Scale (WEANS)* was used to assess Chinese nursing students' willingness in geriatric nursing. This scale was formally developed and validated by Sun et al. (2016). It consists of 21 items, covering four dimensions: behavioural attitudes, subjective norms, perceived behavioural control, and behavioural intentions. Each item is scored on a 5‐point scale (1 = strongly disagree, 5 = strongly agree). Higher scores indicate a stronger willingness to engage in geriatric nursing. The scale shows good internal consistency, with a Cronbach's alpha of 0.795 (Sun et al. 2016). In our study, the Cronbach's *α* value for the WEANS was 0.941.

### 3.6 Data Analysis

Data were analysed using SPSS 23.0 and AMOS 28.0. Descriptive statistics and Pearson correlations were computed first. Second, a confirmatory factor analysis (CFA) was conducted prior to the structural equation model (SEM) to evaluate the psychometric properties of the three measurement instruments in the present sample. Convergent validity was assessed via three criteria (Cheung et al. 2023): (1) all standardised factor loadings ≥ 0.50 and ideally ≥ 0.70; (2) composite reliability (CR) ≥ 0.70; and (3) average variance extracted (AVE) ≥ 0.50. Discriminant validity was assessed via the Fornell‐Larcker criterion and the heterotrait‐monotrait (HTMT) ratio of correlations (HTMT < 0.85). Internal consistency was additionally assessed via Cronbach's *α* (≥ 0.70). The CFA was estimated using maximum likelihood in AMOS 28.0; model fit was evaluated using the same indices used for the structural model.

SEM was employed to verify the interrelationships among the variables. To control for potential confounding, three covariates—age (continuous), gender (male = 0, female = 1), and academic year (second = 0, third = 1)—were regressed on both the mediator and the outcome latent variables. Model fit indices included: *χ* ^2^/df ≤ 3, root mean square error of approximation (RMSEA) ≤ 0.08, Tucker‐Lewis index (TLI) ≥ 0.90, goodness‐of‐fit index (GFI) ≥ 0.90, comparative fit index (CFI) ≥ 0.90, normed fit index (NFI) and Tucker‐Lewis index (TFI) ≥ 0.90 (Hu and Bentler 1999). The bootstrap method was used to confirm the significance of the mediating effect (Wen and Ye 2014). The bootstrap method estimates the distribution of a mediating effect by repeatedly sampling from the original sample and calculates a confidence interval for the mediating effect. If this confidence interval does not include zero, it indicates that the mediating effect is significant. Statistical significance was set at *p* < 0.05 (two‐tailed).

## 4 Results

### 4.1 General Characteristics

A total of 914 students enrolled on the geriatric nursing course. After excluding 18 students who refused to participate and 54 questionnaires with missing critical data, 842 participants were retained for the final analysis. The final sample comprised 555 second‐year students and 287 third‐year students. There were 89 (10.57%) male and 753 (89.43%) female nursing students, with an average age of 19.23 ± 1.51 years.

### 4.2 Nursing Students' PI, Educator Competence Perception and Willingness to Engage in Geriatric Nursing

The total score for PI was 59.77 ± 10.85, with the highest dimension being social modelling (3.89 ± 0.81) and the lowest being social comparison and self‐reflection (3.42 ± 0.67). The total score for educator competence perception was 111.70 ± 19.42, with social ethics scoring highest (3.78 ± 0.70) and practical training scoring lowest (3.66 ± 0.69). The total score for willingness to engage in geriatric nursing was 71.71 ± 11.17, with the highest score in the control dimension (3.50 ± 0.64) and the lowest in the posture dimension (3.36 ± 0.56, Table 1).

**TABLE 1:** Scores of professional identity, educator competence perception and elderly nursing willingness of nursing students (*N* = 842).

Variables	Score range	Score (Mean ± SD)	Item score (Mean ± SD)	Rank
Professional identity	17–85	59.77 ± 10.85	3.52 ± 0.64	
Professional self‐image	6–30	21.69 ± 4.14	3.62 ± 0.69	3
Benefit of retention and risk of turnover	4–20	13.86 ± 2.84	3.65 ± 0.71	2
Social comparison and self‐reflection	3–15	10.27 ± 2.00	3.42 ± 0.67	5
Independence of career choice	2–10	7.17 ± 1.42	3.59 ± 0.71	4
Social modelling	2–10	6.77 ± 1.62	3.89 ± 0.81	1
Educator competence perception	30–150	111.70 ± 19.42	3.72 ± 0.65	
Theoretical course	7–35	26.24 ± 4.88	3.75 ± 0.70	2
Concerns about students	5–25	18.57 ± 3.49	3.72 ± 0.69	3
Career passion	6–30	22.22 ± 4.09	3.70 ± 0.68	4
Practical training	6–30	21.97 ± 4.17	3.66 ± 0.69	5
Social ethics	6–30	22.69 ± 4.18	3.78 ± 0.70	1
Willingness to engage in geriatric nursing	22–105	71.71 ± 11.17	3.42 ± 0.53	
Posture dimension	9–45	30.24 ± 5.06	3.36 ± 0.56	4
Affect dimension	4–20	13.69 ± 2.73	3.42 ± 0.68	2
Control dimension	6–30	20.98 ± 3.85	3.50 ± 0.64	1
Willingness dimension	2–10	6.80 ± 1.53	3.40 ± 0.76	3

### 4.3 Correlation Analysis of PI, Educator Competence Perception and Willingness to Engage in Geriatric Nursing

Correlation analysis (Figure 1) revealed significant positive correlations between educator competence perception and willingness to engage in geriatric nursing (*r* = 0.379, *p* < 0.001), between PI and educator competence perception (*r* = 0.444, *p* < 0.001), and between PI and willingness to engage in geriatric nursing (*r* = 0.413, *p* < 0.001).

**FIGURE 1:**
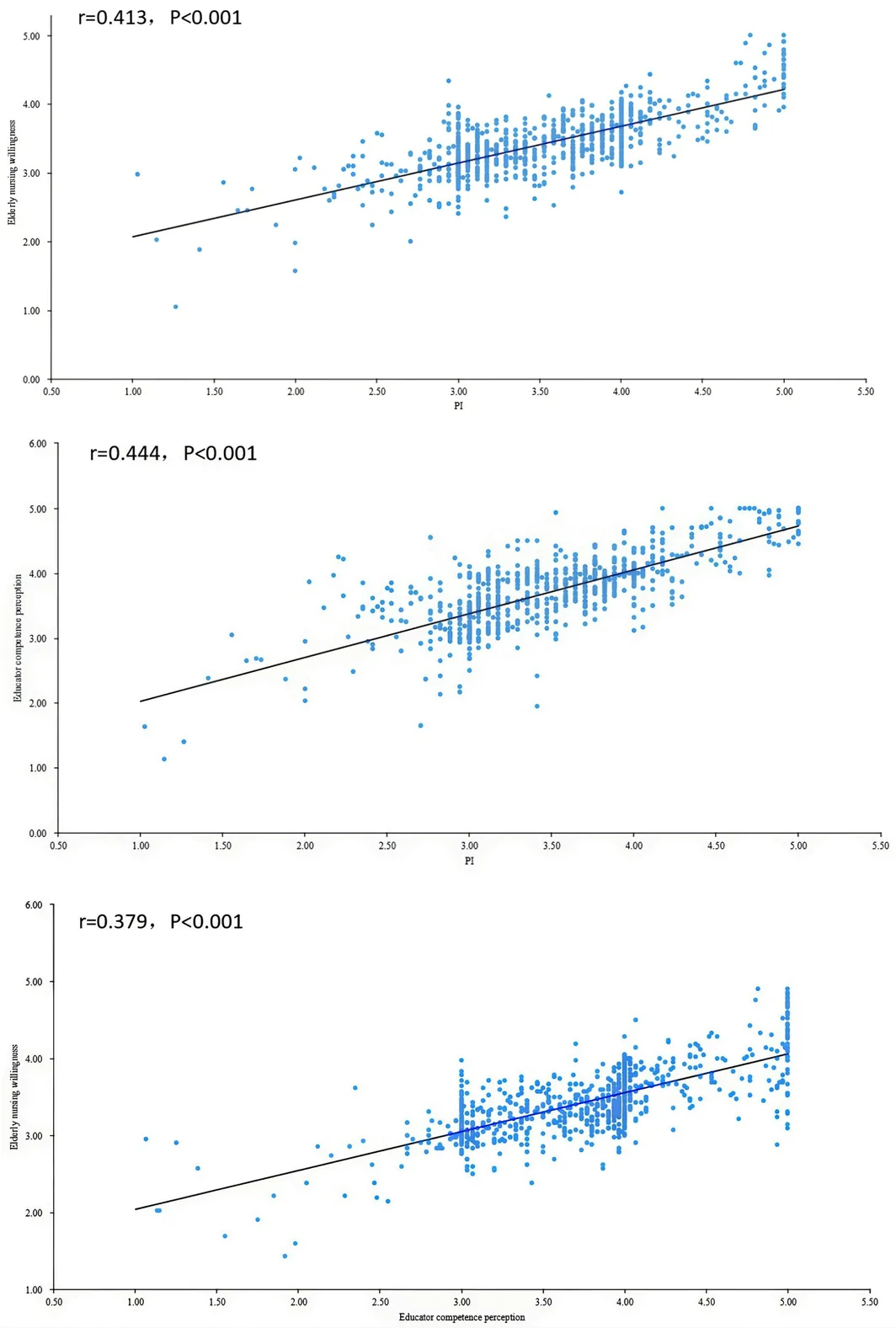
The correlations of professional identity, educator competence perception and willingness to engage in geriatric nursing. PI, professional identity.

### 4.4 Measurement Model Results

Confirmatory factor analysis (CFA) showed acceptable fit for the measurement model (*χ* ^2^/df = 8.29, CFI = 0.953, TLI = 0.943, SRMR = 0.041, RMSEA = 0.093 [90% CI 0.086, 0.100]). All standardised factor loadings were statistically significant at *p* < 0.001. Convergent validity was supported for all three constructs. For educator competence perception, average variance extracted (AVE) = 0.84 and composite reliability (CR) = 0.963. For willingness to engage in geriatric nursing, AVE = 0.61 and CR = 0.86. For PI, AVE = 0.72, which also exceeded the 0.50 threshold. Discriminant validity was supported by the Fornell‐Larcker criterion (the square root of AVE for each construct exceeded all cross‐construct correlations) and by heterotrait‐monotrait (HTMT) ratios (all < 0.85). All three scales showed high internal consistency (Table 2).

**TABLE 2:** Reliability and validity of measurement model (*N* = 842).

Construct	*α*	CR	AVE	√AVE	HTMTmax
PIQNS	0.891	0.925	0.72	0.85	0.73
GeNTS	0.960	0.963	0.84	0.92	0.73
WEANS	0.941	0.858	0.61	0.78	0.72

*Note:* Criteria: *α*, CR ≥ 0.70; AVE ≥ 0.50; square root of AVE > inter‐construct correlations; HTMT < 0.85.

Abbreviations: *α*, Cronbach's alpha; AVE, average variance extracted; CR, composite reliability; HTMT max, maximum HTMT ratio across construct pairs.

### 4.5 Testing the Hypothesised Model

A path analysis using AMOS 28.0 was conducted to explore the relationships among these variables. A structural equation model (SEM) assessed the direct and indirect effects of PI, educator competence perception, and willingness to engage in geriatric nursing. The initial model did not fit well (*χ* ^2^/df = 6.117, *p* < 0.001, GFI = 0.912, TLI = 0.941, CFI = 0.951, NFI = 0.942, RMSEA = 0.078). Post hoc modifications, including adding paths e1–e2, e1–e5, e1–e16, e6–e9, e7–e10, and e8–e10, improved the model fit (*χ* ^2^/df = 2.954, *p* < 0.001, GFI = 0.961, TLI = 0.977, CFI = 0.983, NFI = 0.975, RMSEA = 0.048) (Figure 2). Bootstrapping with 5000 samples confirmed the significance of all path coefficients at the 95% confidence interval (CI), with no CIs including 0 and *Z* > 1.96, indicating that educator competence perception partially mediated the effect of PI on willingness to engage in geriatric nursing.

**FIGURE 2:**
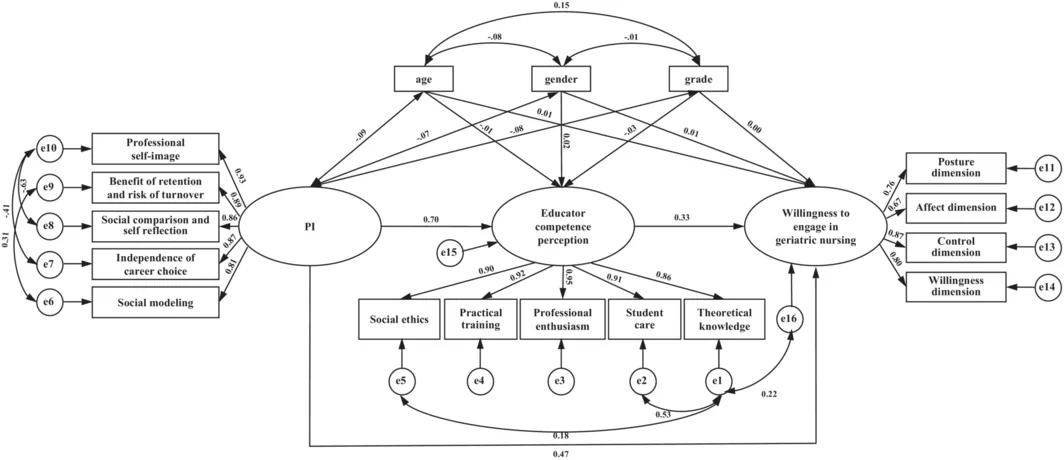
Structural equation model of professional identity, educator competence perception, and willingness to engage in geriatric nursing (*N* = 842), adjusted for age, gender, and grade.

### 4.6 Direct and Indirect Effects

The analysis demonstrated a significant relationship among PI, educator competence perception, and willingness to engage in geriatric nursing. PI was positively associated with educator competence perception (*β* = 0.70, *p* < 0.01), and educator competence perception was positively associated with willingness to engage in geriatric nursing (*β* = 0.33, *p* < 0.01). Educator competence perception mediated the relationship between PI and willingness to engage in geriatric nursing, showing an indirect positive effect. These findings support the hypothesis that educator competence perception mediates the relationship between PI and willingness to engage in geriatric nursing among nursing students (Table 3 and Figure 2).

**TABLE 3:** Bootstrap test of mediating effect.

Item	Structural paths	Standardised effect	Standard error	*Z*	*p*	Bootstrap 95% CI
Total effect	PI→willingness to engage in geriatric nursing	0.700***	0.027	25.926	0.001	(0.642–0.750)
Direct effect	PI→willingness to engage in geriatric nursing	0.470***	0.046	10.217	0.001	(0.379–0.558)
Indirect effect	PI→educator competence perceptio→willingness to engage in geriatric nursing	0.230***	0.046	5.000	0.001	(0.164–0.296)

*Note:* Data are based on 5000 bootstrap samples.

Abbreviations: CI, confidence interval; PI, professional identity.

*** *p* < 0.001.

## 5 Discussion

This study revealed positive associations among PI, educator competence perception, and willingness to engage in geriatric nursing. Notably, educator competence perception partially mediated the association between PI and willingness to engage in geriatric nursing. This suggests that the association between PI and willingness to engage in geriatric nursing may not only be direct but may also be further manifested through students' positive perceptions of educator competence.

This study revealed a significant positive association between PI and nursing students' educator competence perception. Previous research has shown that teachers contribute to the development of nursing students' PI through role modelling (Vabo et al. 2021). Building on this, our findings further suggest that PI may be associated with students' evaluations of educator competence. Students with higher PI typically have a clearer understanding of their professional roles and career values. Therefore, they may pay greater attention to the professional characteristics demonstrated by teachers and consequently develop more positive evaluations of educator competence. This interpretation is also consistent with previous research on the influence of PI on students' perceptions of teachers (Mei et al. 2022). In this study, nursing students in vocational colleges reported moderate to high levels of PI, which were similar to those reported by Liao, Xie, et al. (2024). Nursing students with higher PI have a stronger sense of belonging to and identification with the nursing profession. They are more likely to recognise the professional values conveyed by clinical educators and give more positive evaluations of educators' competence. Existing evidence suggests that fostering nursing students' PI can improve their educational interactions with teaching staff.

This study found a significant positive association between nursing students' PI and their willingness to engage in geriatric nursing, suggesting that higher levels of PI are associated with a stronger willingness to engage in geriatric nursing. This finding is broadly consistent with previous research (Li et al. 2024; Moll‐Jongerius et al. 2023). In this study, the willingness to engage in geriatric nursing was at a moderate level. This finding was in line with previous studies, which reported limited willingness among nursing students to choose geriatric nursing as a career (Wang et al. 2024). However, some differences remained across studies, which may be related to differences in study populations, educational stages, and measurement instruments (Dai et al. 2021). PI is an important foundation for nursing students to develop professional values and clarify their professional roles (Chen et al. 2026). Students with higher levels of PI typically have a clearer understanding of their nursing roles and are more likely to view nursing as an important part of their future career (Xu et al. 2024). Therefore, higher levels of PI may lead nursing students to recognise the professional value of geriatric nursing and be more likely to demonstrate a stronger willingness to engage in geriatric nursing (Yang et al. 2025).

This study also found a significant positive association between educator competence perception and nursing students' willingness to engage in geriatric nursing, which is consistent with the findings of Garbrah et al. (2021). Previous research has shown that the professional competence of geriatric nursing educators can positively influence students' willingness to engage in geriatric care. Compared with teachers' personal characteristics, nursing students place greater value on their professional competence (Labrague et al. 2020). Students with more positive evaluations of geriatric nursing educators' competence are more likely to accept knowledge related to geriatric care, and consequently have a stronger willingness to engage in geriatric nursing. Therefore, nursing institutions can support students in developing positive learning experiences and career perceptions related to geriatric nursing by strengthening geriatric nursing educators' professional competence.

One of the most important findings of this study was that educator competence perception partially mediated the association between PI and willingness to engage in geriatric nursing. This finding suggests that the association between PI and willingness to engage in geriatric nursing may not only involve a direct effect but may also be further reflected through students' positive perceptions of educators' professional competence (Garbrah, Kankkunen, and Välimäki 2020). Students with higher levels of PI typically have clearer professional standards and may therefore pay greater attention to teachers' professional competence, leading to more positive educator competence perceptions (Li et al. 2024). Meanwhile, positive educator competence perceptions may provide students with more positive learning experiences. They may also help students understand the significance of geriatric nursing (Garbrah et al. 2021). As a result, students may develop greater willingness to engage in geriatric nursing (Garbrah, Kankkunen, and Välimäki 2020). Therefore, educator competence perception may constitute an important educational pathway through which PI is translated into willingness to pursue a career in geriatric nursing. Notably, this study observed partial rather than full mediation, suggesting that other mechanisms not examined in this study may also underlie the association between PI and willingness to engage in geriatric nursing. Therefore, nursing education practice should not focus solely on developing students' PI but should also emphasise teachers' professional competence. Providing positive learning experiences in geriatric nursing may help students develop clearer career perceptions and further promote their choice of geriatric nursing as a career (Vabo et al. 2021; Garbrah, Kankkunen, and Välimäki 2020).

## 6 Limitations

Several limitations existed in this study. First, 89.43% of the participants were female, resulting in an imbalanced gender distribution that may limit the applicability of the findings to male nursing students. Second, this study used a non‐probability sampling method and was conducted at a single site in China. Therefore, the external validity of the findings may be limited, and caution is warranted when generalising the findings to different regions and educational levels. Future studies could use multicentre random or stratified sampling and include more diverse groups of nursing students. Third, data were collected using self‐report measures, which may have introduced reporting bias. In addition, the cross‐sectional design limits the ability to establish causal relationships. Future longitudinal or interventional studies could further examine whether educator competence perception constitutes a modifiable pathway between PI and willingness to engage in geriatric nursing. Such studies could provide stronger empirical evidence for the design of nursing education interventions.

## 7 Conclusion

This study showed that both PI and educator competence perception were positively associated with nursing students' willingness to engage in geriatric nursing. Educator competence perception also partially mediated the association between PI and willingness to engage in geriatric nursing. This finding suggests that the association between students' PI and their choice of geriatric nursing may be partly reflected through their positive perceptions of educators' competence. In addition to fostering students' PI, nursing education practice should also emphasise the professional competence of geriatric nursing educators. This may support students in developing positive learning experiences and career perceptions related to geriatric nursing. Given the cross‐sectional design of this study, future longitudinal and interventional studies are needed to further validate these associations and their potential mechanisms.

## Author Contributions

**Ting Liao:** investigation, data curation, formal analysis, software, validation, writing – original draft, writing – review and editing. **Jinmei Zou:** conceptualization, methodology, writing – review and editing, project administration. **Min Wang:** investigation, methodology, data curation. **Yuqing Song:** methodology, project administration, formal analysis, writing – original draft. **Li Cai:** investigation, data curation. **Jin Yin:** methodology, data curation, formal analysis. **Yao Chen:** conceptualization, project administration, writing – review and editing. **Yating Wang:** methodology, project administration, writing – review and editing.

## Funding

Research Project of Sichuan Vocational College of Health and Rehabilitation (CWKY‐2024Z‐05). Zigong Health Humanities Research Center (Grant No. JKRWY25‐21).

## Ethics Statement

The study was conducted in accordance with the Declaration of Helsinki and approved by the Zigong First People's Hospital Committee (No. Ethics (Research) No. 129 of 2023).

## Conflicts of Interest

The authors declare no conflicts of interest.

## Data Availability

The data presented in this study are available on request from the corresponding.
